# Structure and Properties of ZrO_2_–20%Al_2_O_3_ Ceramic Composites Obtained Using Additive Technologies

**DOI:** 10.3390/ma11122361

**Published:** 2018-11-23

**Authors:** Vladimir Promakhov, Alexander Zhukov, Yana Dubkova, Ilya Zhukov, Sergey Kovalchuk, Tatyana Zhukova, Andrey Olisov, Viktor Klimenko, Nadezhda Savkina

**Affiliations:** 1 National Research Tomsk State University, 36 Lenin Ave., 634050 Tomsk, Russia; vvpromakhov@mail.ru (V.P.); zhuk_77@mail.ru (A.Z.); gofra930@gmail.com (I.Z.); kovalchuk.s.v@inbox.ru (S.K.); zhu-tanya@yandex.ru (T.Z.); kobis@bk.ru (A.O.); science@klimenko.team (V.K.); savkina@ftf.tsu.ru (N.S.); 2 Institute for Problems of Chemical & Energetic Technologies of the SB RAS, 1 Sotsialisticheskaja Str., 659332 Biysk, Russia; 3 Company “Nanoceramics”, 20 Frunze Ave., 634029 Tomsk, Russia

**Keywords:** additive technologies, ceramic materials, compositional structure, mechanical properties

## Abstract

This investigation focused on obtaining samples from ceramic composite materials, based on the ZrO_2_–20%Al_2_O_3_ system, using the additive layer-by-layer fusion technology for thermoplastic systems. The structure and phase composition of the initial powders were studied, experimental samples were produced, and the structure and properties of the experimental samples that were obtained using additive technologies were analysed. The measured static bending strength of the samples was 450 ± 70 MPa, microhardness was 14 GPa, and the elasticity modulus was 280 ± 25 GPa. The strength of these samples are slightly inferior to that of similar materials, obtained using Ceramic Injection Molding technology because our samples were characterised by the residual porosity of about 15%.

## 1. Introduction

The development and fabrication of advanced ceramics for high-technology applications, such as automobile, aerospace, defense, power engineering, environmental, and biomedical, are scientifically and technologically challenging tasks. Examples of state-of-the-art and potential applications of advanced ceramics have been described in the literature [1]. The demand for ceramic materials depends primarily on the growth of end-user markets. However, technological innovations have continually improved the performance and efficiency, stimulating the extended use of advanced ceramics in key sectors of the Russian economy [2]. Many industrial applications of advanced ceramic materials depend on the technological availability of the fabrication of three-dimensional (3D) ceramic items that have the required geometry [3,4]. Geometry is of special significance in the field of ceramics science, and post-processing is time-consuming and expensive, and normally requires the use of diamond tools. Thus, in many cases, post-processing accounts for up to 80% of the total manufacturing costs of a ceramic item [5]. Also, for small-scale production and prototyping, the costs of modelling and molding are critical in the cost structure. This has led to the emergence of new technologies, such as additive manufacturing (AM). These demand and importance of these new technologies are increasing.

According to the general classification of additive technologies (EN ISO/ASTM 5291:2107 and ISO/ASTM 52900), there are two different categories of AM processes: (1) single-step processes (also called “direct” processes), in which parts are manufactured in one operation, wherein the main geometric shape and the main material properties of the required product are achieved simultaneously; and (2) multi-step processes (also called “indirect” processes), during which parts are produced in two or more operations. The first operation, in general, provides the main geometric shapes, and the next operation unites the part into the assumed basic material properties [1,2,3,4,5,6,7]. Most of the AM processes for creating ceramics are multistage (indirect) processes. In such processes, binder materials are used to make ceramic pastes. These binders are then removed by heat treatment in a furnace.

Single-stage processes for the formation of ceramics directly supply energy to the construction site (direct energy deposition) and the melting of the material in a pre-formed layer (powder bed fusion). The latter process includes selective laser melting (SLM) and single-stage selective laser sintering [8]. Attempts to use powders of refractory compounds (ceramic powders) in these technologies have demonstrated that it is impossible to obtain a defect-free structure due to the specificity of the atomic interaction in ceramics. Thus, for ceramics, using multi-stage technologies is common [9].

One of these technologies is based on the formation of ceramics by deposition of ceramic paste [10] using extruders in stages to create 3D structures [11,12]. Other synonyms for these technologies, which can lead to confusion and do not usually mean fused deposition of ceramics (FDC), include: extrusion freeform fabrication (EFF) [13,14], Direct Ink Writing (DIW) [11], suspension application [15], Bioplotting, [16] Rapid Prototyping Robotic Dispensing (RPRD), Microextrusion Freeforming (MF), and Robocasting (RC) [17].These similar technologies differ slightly in terms of process details. To avoid confusion, we clarify that in the present work, the ceramic suspension is deposited on the substrate at ambient temperature, which is closer to Robocasting technology.

The geometric design possibilities combined with the development of new materials provides a unique opportunity to reduce the manufacturing time and production cost of components with the required properties. This is one advantage of additive technologies. However, dimensional accuracy and surface cleanliness are still the most important limitations of additive manufacturing. For example, it is difficult to achieve accurate layer deposition by distributing thin powder layers during 3D printing, or to ensure uniform re-coating of a new layer with a high-viscosity ceramic slurry in Stereolithography (SLA) [18]. Rough surfaces on the produced parts are the result of the “ladder” effect, which are due to the cutting of the contoured surface [19]. Texture can be improved by reducing the thickness of individual structural elements or by subsequent secondary operations, such as grinding or polishing. However, these stages of work increase the time and cost of production. Unfortunately, each process has its own unique drawbacks. For example, SLA demonstrates the best surface cleanliness among the currently available technologies, but it uses a very expensive and limited range of raw materials (photosensitive resins), while additional care must be taken due to the environmentally hazardous solvents that are used to clean the parts [20]. Robocasting and Material Extrusion with a piston provides less accurate parts, but allow the use of a wide range of raw materials (in principle, any material that is available in powder form). In addition, 3D printing allows the control of not only the shape of ceramic parts but also the composition, microstructure, and properties of the component. Thus, obstacles still exist regarding the introduction of these technologies into production processes. Although significant progress has been made for metal materials, for ceramic materials, the most difficult limitations for AM technologies are those imposed by the choice of material for each AM method, and aspects that consider the internal structure of the material after annealing and sintering. In this regard, the development and study of the processes of formation of the structure and properties of ceramic composite materials obtained with the use of various technologies of additive production are relevant. The main purpose of this work was to study the possibility of obtaining composite materials using additive technologies and to study their properties.

## 2. Materials and Methods

**Figure 1:**
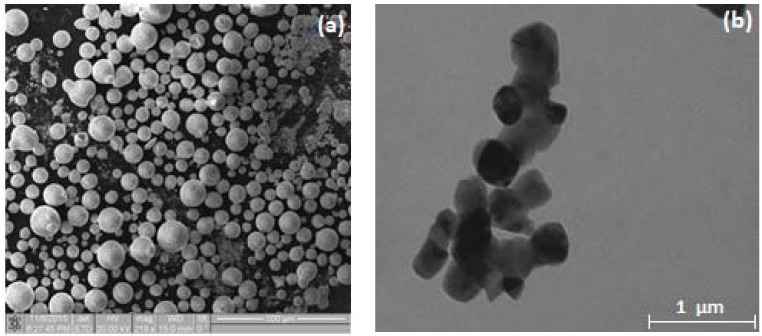
Images of ZrO_2_(3%Y_2_O_3_) + 20%Al_2_O_3_ powders: (**a**) general view of powders and (**b**) image of nanoparticles of which compose the granules.

To date, the specialists at Tomsk State University (TSU), Siberia, Russia have developed an method of additive printing complex profile items based on the principles of Material Extrusion with a piston implementing the deposition of finely dispersed thermoplastic systems (i.e., technology compositions of powders and thermoplastic substances) [2,3,4,5]. The specialists from TSU created a 3D printer that can work with thermoplastic pastes (suspensions) that are based on ceramic powders. The entire workflow includes the preparation of initial materials, material deposition through the nozzle, and a post-processing stage. Post-processing includes the removal of the binder and a sintering process. The samples in the present research were fabricated from powders produced by Tosoh Corporation (Tokyo, Japan). Using scanning electron microscopy (SEM), Figure 1 shows the images obtained for the ZrO_2_(3%Y_2_O_3_) + 20%Al_2_O_3_ powders. The powders consisted of granules with an average size of 40–80 µm (Figure 1a). The granules were comprised of nanoparticles of approximately 100 nm in size (Figure 1b). The particles had a rather narrow size distribution.

It has been established that powders consist of agglomerated nanostructure particles with an average agglomerate size of 30–50 µm. This powder structure meets the general requirements for the fabrication of high-solids suspensions: the nanostructure facilitates intensive sintering, and nanoparticle agglomerates reduce total specific area, thus leading to the decreased content of the binder in the suspension [21,22,23,24]. The specific area, measured via the low-temperature adsorption of nitrogen vapors (multi-point BET (Brunauer-Emmet-Teller)), was 7.83 m^2^/g. The phase composition of the powder is represented by ZrO_2_ phases: the tetragonal and the monoclinic phase of ZrO_2_ and the alpha phase of aluminum oxide.

The powders were used to prepare thermoplastic pastes with different binder contents. A mixture of paraffin (melting point 48 °C) and wax (melting point 58 °C) was used as a binder in a paraffin/wax proportion of 95:5. The parameters of the viscosity of the system were determined using a rotational viscometer (Rotary viscometer (complex) US-2200A; Xieli International Trading Co., Ltd., Wanchai, China). We established a dependency between the content of the binder and the material viscosity, and a dependency of viscosity on the temperature in the range of 60 to 100 °C. We discovered that at a binder content between 28 and 32 wt % and in a temperature range of 90 to 100 °C, the suspension viscosity was not more than 25–40 Pa∙s. (at shear rate 100 s^−1^ and constant speed). 

The powders were mixed with the binder at 80 °C in a –2200A mechanical mixer overhead stirrer (Xieli International Trading Co., Ltd., Wanchai, China) equipped with a DC-1006 Low-Temp Thermostat (Scientz, Ninbo, China). We varied the content of powders in the binder between 20 and 70 wt %. We established that when the amount of powder in the binder was increased to above 60 wt %, the viscosity of the suspension increased critically. The viscosity of the suspension, when the powder content in the binder was 60 wt %, was 10–15 Pa∙s. Thus, to obtain denser ceramics and to maintain manufacturability, a composition with the powder content of 60 wt % in the binder was selected.

The initial structure of the powders and the fabricated composition materials were researched using scanning electron microscopy with a Quanta 200™ 3D microscope (FEI Company, Thermo Fisher Scientific, Waltham, Massachusetts, USA). For SEM, sample thin sections were prepared, then a thin layer of silver was deposited on the surface of ceramics, and then studies were performed. The parameters of the initial powders were researched by X-ray diffraction on a Shimadzu XRD 6000 X-ray diffractometer (Shimadzu Corporation, Tokyo, Japan) using filtered CuKα radiation. The specific density of the powders was measured via low-temperature adsorption of nitrogen. The density of the obtained composites was measured using hydrostatic weighing. Vickers hardness was measured on a table top NHT-TTX nanoindentation tester (CSM Instruments, Needham, Massachusetts, USA) at the maximum load of 100 mN, maximum indentation depth of 2100 nm, and load rate of 200 mN/min. Here, the loading time was 15 s. Mechanical tests of the composites were performed on an Instron^®^ 3369 universal testing machine (Instron^®^, ITW Test & Measurement group, Norwood, Massachusetts, USA), at room temperature (T = 25 °C), atmospheric pressure, and loading rate 0.3 mm/s. For testing, samples with 100% filling were used. Each test was performed on five samples (sample sizes 5 × 5 × 40 mm). For polishing, samples were polished with an EcoMet™ 250 Grinder Polisher (Buehler, Lake Bluff, Illinois, USA). The measurement of the specific surface was performed using Sorbi^®^-M (http://meta-sorbi.ru/products/sorbim/, Meta Ltd., Novosibirsk, Russia). The operation of the device is based on the measurement of specific surface according to the multipoint BET method.

## 3. Equipment

**Figure 2:**
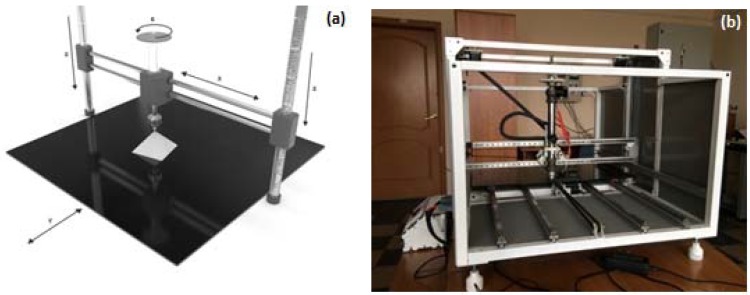
Three-dimensional (3D) printer (**a**) scheme and (**b**) general view.

For the production of the ceramic blanks, a predetermined shape was used in the original system, with a floating dispenser and by moving the substrate (Figure 2a,b).

**Figure 3:**
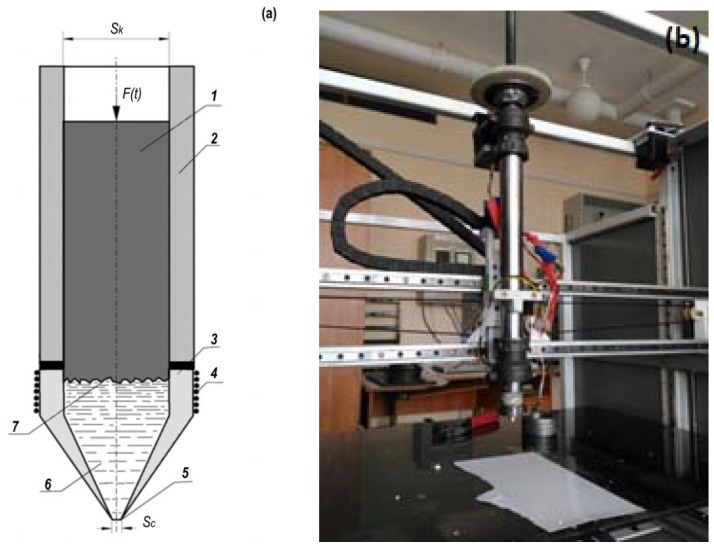
Extruder (**a**) scheme and (**b**) general view.

An original extruder was developed for controlled the feeding of ceramic paste. Figure 3a,b present a general view of the developed extruder and its scheme. The printing process was implemented as follows. The feedstock material (1) was placed in a cylindrical container (2). Part (3) of the container was heated with a spiral (4) from the power supply. When applying pressure (F), a part of the ceramic paste (6) was fed through the extruder nozzle (7) with a hole diameter of Sc ~0.6 mm.

The feed force of the rod on the movable head is determined by a given dependence of the flow rate of the thermoplastic suspension over the time *G*(*t*), which is calculated based on the specific geometry of the product. The flow rate through the narrowing device-nozzle is determined by the following ratio [1]:

(1) $$G(t)=\varphi S_{c}\sqrt{2\rho \Delta p(t)}\;$$

where φ is the mass-flow coefficient of the nozzle of the movable head, *S_c_* is the minimum area of the nozzle of the movable head (m^2^), Δ*p*(*t*) = *p*(*t*) *− p_a_* is the pressure drop (Pa), *p_a_* is the atmospheric pressure (Pa), and *p(t)* is the melt pressure in the movable head (Pa).

From the ratio in Equation (1), *p*(*t*) can be determined:

(2) $$p(t)=p_{a}+\frac{1}{2\rho }{\left[\frac{G(t)}{\varphi S_{c}}\right]}^{2}\;$$

To create a pressure *p*(*t*) in the movable head, it is necessary to create a rod feed force *F*(*t*) equal to:

(3) $$F(t)=p(t)\cdot S_{k}\;$$

From Equations (2) and (3), the ratio in Equation (4) can be followed:

(4) $$F(t)=\left[p_{a}+\frac{1}{2\rho }{\left(\frac{G(t)}{\varphi S_{c}}\right)}^{2}\right]S_{k}\;$$

The developed method is implemented as follows. The computer automated design (CAD) system designs the required part geometry, which is then converted into commands to control the movement of the head and the feed force of the rod. The cylindrical rod is placed in a container and the control program is run. To generate a G-code, we used Slic3r, which is a G-code generator for 3D printers [25]. The layer-by-layer melting of the ceramic blank with a binder occurs in the local heating zone, while the melt is metered out through the nozzle of the movable head.

**Figure 4:**
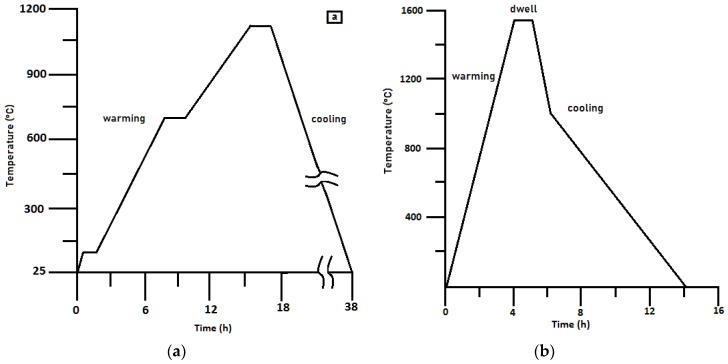
Thermal treatment (**a**) debinding, (**b**) mode of high-temperature sintering of ceramic samples.

The construction of a given geometry of the product from a thermoplastic suspension is carried out using the layer-by-layer deposition method. The binder was removed in an air oven at a temperature of 1100 °C, and the heating rate was not more than 50°/h. To form the final part, the resulting billet was subjected to cooling, using debinding (Figure 4a,b) to remove the technological bunch, followed by high-temperature sintering according to the mode shown in Figure 4. Sintering was carried out in air.

### Selection of Modes for Printing

**Figure 5:**
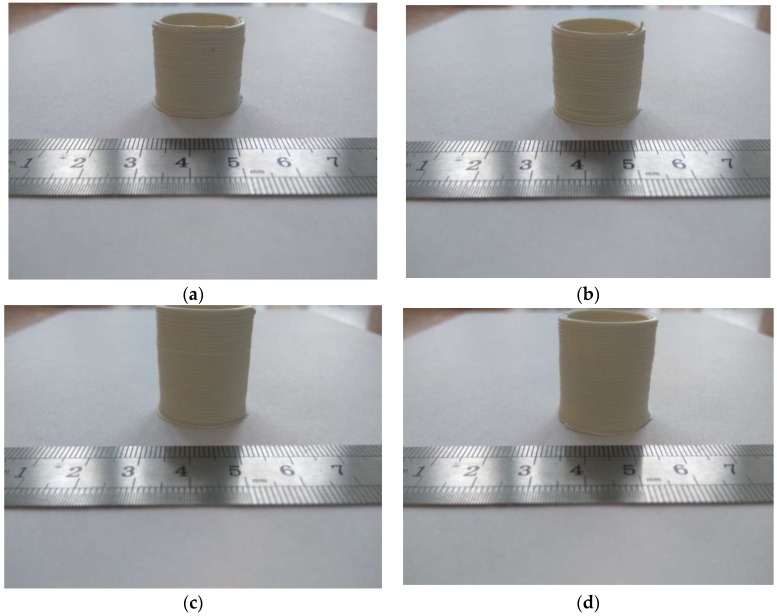

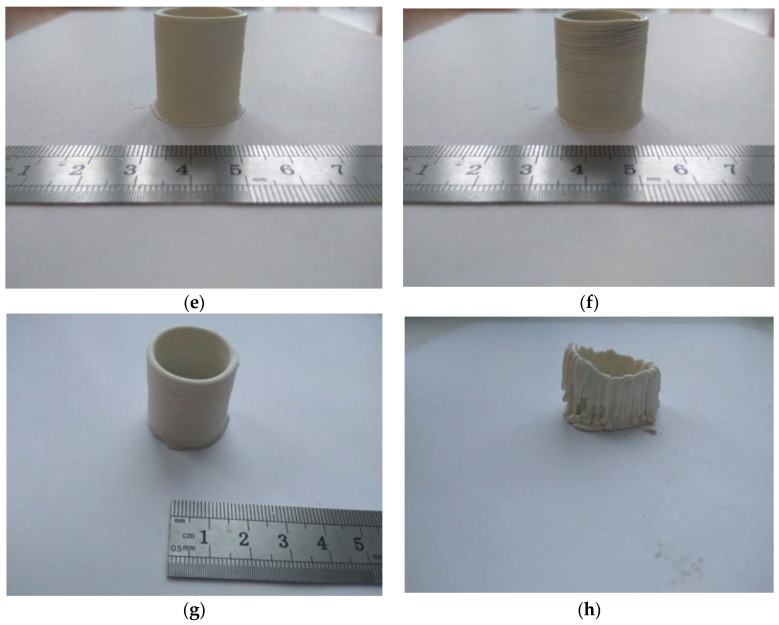
Images of the printing process of ceramic samples at a constant printing speed and using different temperatures for the hot zone of the movable head. (**a**) Sample No. 1: Temperature 60 °C, wall thickness 3 mm; (**b**) Sample No. 2: Temperature 61 °C, wall thickness 3 mm; (**c**) Sample No. 3: Temperature 62 °C, wall thickness 3 mm; (**d**) Sample No. 4: Temperature 63 °C, wall thickness 3.15 mm; (**e**) Sample No. 5: Temperature 64 °C, wall thickness 3.6 mm; (**f**) Sample No. 6: Temperature 65 °C, wall thickness 3.7 mm; (**g**) Sample No. 7: Temperature 66 °C, wall thickness 3.9 mm; and (**h**) Sample No. 8: Temperature 67 °C.

To test the technological modes of obtaining products from ceramics with complex shape, a number of research experiments were carried out, in which the supply temperature of ceramic paste was varied. In all experiments, the following fixed parameters were established: the print speed was 2.5 mm/s, the diameter of the samples was 18.5 mm, and the diameter of the Spinneret was 0.8 mm. For all samples, the material for the printing paste was based on the powders of the system ZrO_2_(3Y_2_O_3_) − 20%Al_2_O_3_. Figure 5 shows a number of images of the printing process of ceramic samples at a constant printing speed and with different temperatures for the hot zone of the movable head. The print quality was recorded visually and determined by the wall thickness of the samples.

**Figure 6:**
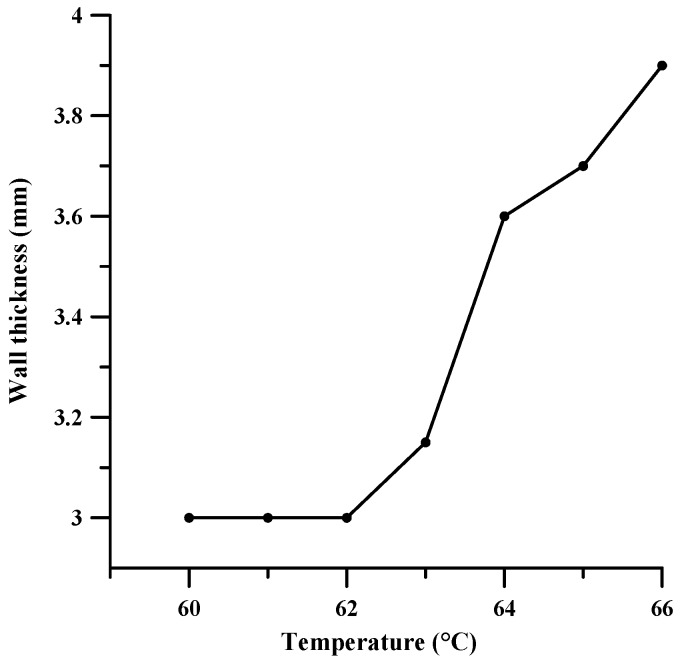
The dependence of wall thickness on temperature printing.

The dependence of wall thickness on temperature is shown in Figure 6.

We found that the increase in the temperature of the hot zone of the movable head, in the range of 60 °C to 67 °C, critically affected the quality of the printed samples. Starting from the printing temperature of 62 °C, the wall thickness of the samples increased due to the large spread of the paste on each layer of the samples. At 66 °C, the wall thickness of the samples reached its critical thickness and at a printing temperature of 67 °C, each subsequent layer melted the previous layer, which made it impossible to print samples. Also, at temperatures above 65 °C, the quality of the samples decreased due to problems arising in the printing process including: jamming of the workpiece in the extruder and excessive material supply. Based on the quality of the samples at the optimum temperature, the selected value was 62 °C.

## 4. Results and Discussion

**Figure 7:**
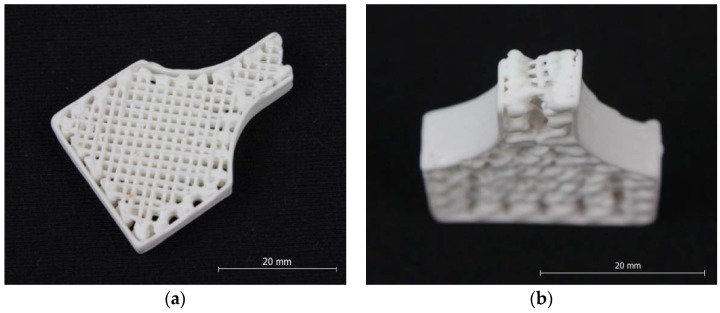
Ceramic samples fabricated by three-dimensional (3D) printing with a fill factor of 50%: (**a**) view from above and (**b**) side view.

Printing was performed using a recently developed process [3] and according to the standard routine. The original geometry was selected for fabricating sample items with the required geometry (Figure 7a,b). 3D models were translated into Computer Numerical Control (CNC) commands using specialised Slic3r software. In the Slic3r software [25], the following printing parameters were selected for sample fabrication: nozzle output aperture diameter of 0.7 mm, print layer thickness of 0.8 mm, internal fill factor of 50%, printing temperature of 62 °C, and printing speed of 20 mm/min.

**Figure 8:**
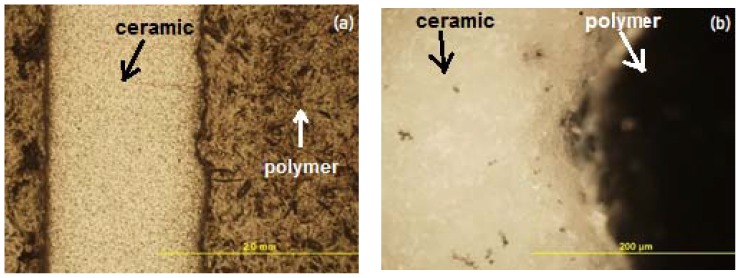
Images of the butt-ends of the fabricated samples using the developed additive printing method: (**a**) 200× magnification image; (**b**) 1000× magnification image.

We used an optical microscope Olimpus GX (Olympus Corporation^©^, Tokyo, Japan) to analyse the structure of the layers that were obtained. We established that the porosities of the samples did not exceed 12%. Using an optical microscope, we determined that the microstructure of the samples was uniform, monolithic, and without cracks (Figure 7a,b). After a visual inspection, no delamination between layers was noticed. As shown in Figure 8, the polymer was used to press and polish samples.

**Figure 9:**
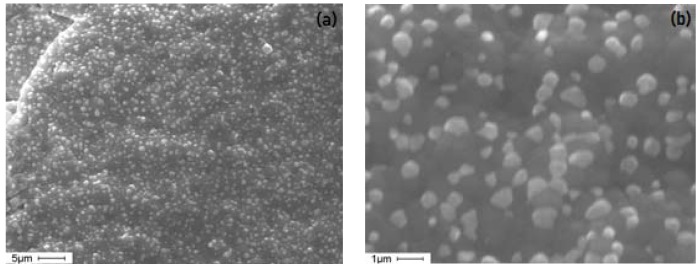
Images of the structure of the ceramics, generated on a scanning electron microscope: (**a**) 3000× magnification image; (**b**) 8000× magnification image.

The granular structure of the samples was researched on the Quanta 200 3D scanning electron microscope (FEI Company, Hillsboro, Oregon, USA). The images of the ceramic samples structure are shown in Figure 9a,b.

**Figure 10:**
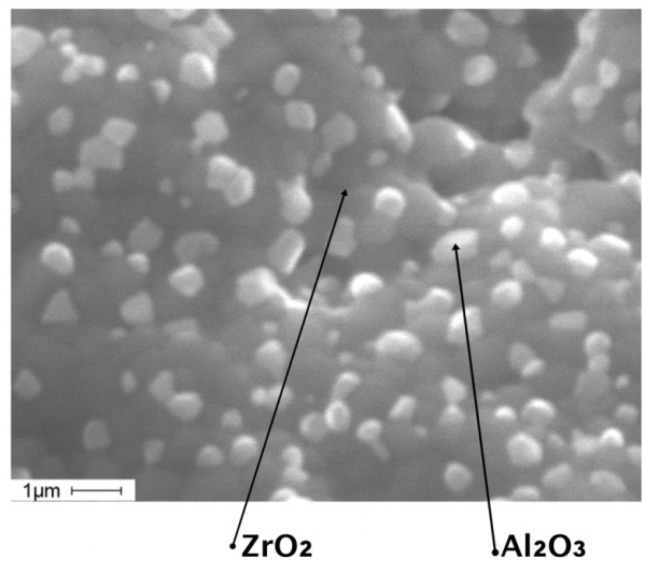
Image of the structure of ZrO_2_(Y_2_O_3_)-20%Al_2_O_3_ ceramics obtained after high-temperature sintering.

Figure 10 shows the structure of ZrO_2_(Y_2_O_3_) + 20%Al_2_O_3_ ceramics obtained after high-temperature sintering. We determined that the average grain size was 700 nm. The average grain size of aluminum oxide was 650 nm. According to the literature [26,27,28], this average grain size provides optimal conditions for the manifestation of the transformation hardening effect in a ZrO_2_(Y_2_O_3_) + 20%Al_2_O_3_ system. Further, aluminum oxide grains were clearly discernible inside the structure, and those play the role of the strengthening phase.

Thus, a method of fabricating ceramic items using digital technologies was laboratory tested; the microstructures of the samples that were fabricated by the additive technology were researched; and the hardness properties of ZrO_2_(Y_2_O_3_) + 20%Al_2_O_3_ ceramics were tested. The measured static bending strength (three-point bending strength test) of the samples was 450 ± 70 MPa, microhardness (HV) was 14 GPa, and the elasticity modulus (E) was 280 ± 25 GPa (the elasticity modulus was determined from the diagram of aggression). The strength values are slightly inferior to the strength of similar materials produced by CIM technology, because our samples are characterised by a residual porosity of about 15%. Porosity was determined by hydrostatic weighing. The residual porosity arises due to the temperature of sintering being 1500 °C. When the temperature rises to 1600 °C, these materials have a dense structure [29,30].

Comparison with the data from the literature showed that the levels of properties of the obtained samples correspond to those of samples from similar materials that were obtained by pressing. For example, J.K. Daguano et al. [29] studied the properties of composites ZrO_2_(Y_2_O_3_) + 20%Al_2_O_3_, obtained by pressing and sintering. The authors established that the hardness of the samples was 1400 HV. C. Santos et al. [30] reviewed the properties of the composites that are more widely used at different sintering temperatures. Ceramic samples were obtained by pressing and sintering at 1500, 1550, and 1600 °С for 120 min. They established that the bending strength after sintering the composites at a temperature of 1600 °C for 2 h was 690 MPa.

The extremely high melting point of many ceramic materials makes it difficult to manufacture parts using additive technologies, compared to metals and polymers. Since ceramics cannot be cast or processed easily, 3D printing provides a big improvement in geometric flexibility [31,32]. We report the possibility of obtaining samples with complex shapes from ceramic materials using nanopowders. Using our approach, it is possible to produce various ceramic compositions, including materials that are difficult to form using sintering powders, such as Al_2_O_3_, ZrO_2_, Si_3_N_4_, and SiC ceramics, obtained by plasma-chemical synthesis [33]. In this article, we focused on structures made of composite materials ZrO_2_(Y_2_O_3_) + 20%Al_2_O_3_. Such ceramic materials are of interest for the core of lightweight ceramic sandwich panels for high-temperature applications, for example, in hypersonic vehicles and jet engines [34,35]. 3D printing with ceramic materials will create opportunities for the manufacture of parts that have complex shapes that are resistant to aggressive environments and high temperatures, for example, in microelectromechanical systems (MEMS) or in thermal protection systems.

## 5. Conclusions

Composite materials are widely used in many fields, such as construction, electronics, and medicine [35,36]. They are extremely interactive with other materials and their mechanical characteristics can be enhanced by adding nanoparticle [37] or fiber [38] fillers. Therefore, the possibility of combining the characteristics of ceramic composite materials with 3D printing technology is promising for future applications in materials science. As a result of this research, we showed that, with the application of Material Extrusion with piston technology, it is possible to obtain samples with complex geometries from oxide ceramic materials. We found that after sintering the obtained samples, based on the ZrO_2_-20%Al_2_O_3_ system, the controlled layer height was approximately 600 μm. We found that the internal structure of the ceramic is monolithic without distinct boundaries between the horizontal and vertical (wall) layers of material in the samples. This allows us to state that the developed 3D method for molding products from technical ceramics enables the manufacturing of products for structural and functional purposes. The strength and hardness of additive ceramic structures are not inferior to the parameters of the samples obtained by traditional injection molding technology.

The strength of the ceramic samples that were obtained using additive technology was 450 ± 70 MPa, which was guaranteed by a low residual porosity in the microstructure of the material. Microhardness was 14 GPa, and the elasticity modulus was 280 ± 25 GPa. These samples are characterised by a residual porosity of approximately 15%.
